# Diagnosis of Carcinoma of the Breast in Its Early Stages

**Published:** 1897-03

**Authors:** 


### DIAGNOSIS OF CARCINOMA OF THE BREAST IN ITS EARLY STAGES.

Dr. A. Marmaduke Shield, in a clinical lecture at St. George's Hospital, goes over this subject very carefully and clearly. The question involved in the accurate diagnosis of this condition is such an important one that a careful study of the symptoms as observed in the large experience of Shield is valuable.

He states that out of seven hundred and fifty cases of breast-diseases of all kinds at St. George's Hospital, between the years 1875 and 1895, three hundred and fifty-three, or about one-half, were carcinomatous.

In making the examination he advises that the thorax be completely undressed. The examination should be made with the flat of the fingers, and not by roughly pinching up a portion of the gland. The following points are to be noted:

1. The age; the most likely age is between forty and fifty-five.

2. The character of the pain, which is neuralgic, not throbbing and is not accompanied by heat or redness.

3. The onset is insidious.

4. The contour is rarely globular; usually it is obtuse or irregular.

5 The hardness is stony, and is one of the most striking and reliable characteristics of scirrhus.

6. The growth does not move in the breast substance, but moves with it.

7. Retraction of the nipple is an important symptom, but it may be absent.

8. The dimpling of and the pig-skin-like condition of the skin are of the utmost value.

9. The presence of enlarged glands is important, but their absence is not proof that the disease is innocent.

In cases of doubt the author is strongly in favor of exploratory incision. He believes there are peculiarities of structure or of growth in certain individuals which may be sufficiently pronounced to become hereditary, and thus a predisposition to carcinoma may be found in some families. He calls attention to the fact that certain obscure conditions coming on in elderly women, such as an insidious pleurisy, hydrothorax, supposed "rheumatic" pains about the thorax or bones, or, of still greater importance, spontaneous fracture, or severe pains in the spine, terminating in paraplegia, may be due to cancer, and that in all such cases the breast should be carefully examined, as the discovery of scirrhus nodule may explain a very mysterious illness.

He insists upon a very careful examination of the breast, lungs, and abdomen, especially of the liver, for secondary growths, as having important bearings on the question of operation.—*International Medical Magazine.*

